# Hydrido(prolinato-κ^2^ *N*,*O*)tris­(tri­methyl­phosphane-κ*P*)iridium(III) hexa­fluorido­phosphate

**DOI:** 10.1107/S1600536814001834

**Published:** 2014-01-31

**Authors:** Joseph S. Merola, Christopher P. Roy

**Affiliations:** a Department of Chemistry, Virginia Tech, Blacksburg, VA 24061, USA; b Department of Chemistry, Duke University, Durham, NC 27708, USA

## Abstract

The title complex, [Ir(C_5_H_8_NO_2_)H(C_3_H_9_P)_3_]PF_6_, has two independent anion–cation pairs in the asymmetric unit. The geometry about each Ir^III^ atom is pseudo-octa­hedral with a meridional arrangement of the P(CH_3_)_3_ ligands, *N*,*O*-bidentate coordination of prolinate and a hydride ligand *trans* to the prolinate N atom. The independent Ir^III^ moieties are joined by N—H⋯O hydrogen bonds and the N—H⋯O bonding motif continues throughout the structure, creating an extended chain parallel to the *c-*axis direction. The methyl groups of one P(CH_3_)_3_ ligand are rotationally disordered over two sets of sites in a 0.62 (2):0.38 (2) ratio.

## Related literature   

For the valine structure analogous to the proline structure described herein, see: Roy *et al.* (2006 ▶). For a Cp*Ir complex with proline and a *t*-butyl­ethynl ligand, see: Carmona *et al.* (2000 ▶). For a Cp*Ir complex with proline and a chloride ligand, see: Carmona *et al.* (2012 ▶). For the preparation of [Ir(COD)(PMe_3_)_3_]Cl, see: Frazier & Merola (1992 ▶). For a selection of amino acid complexes in general, their structures and their extended lattice features, see: Urban *et al.* (1996 ▶); Shimazaki *et al.* (2009 ▶). For a description of the Cambridge Structural Database, see: Allen (2002 ▶).
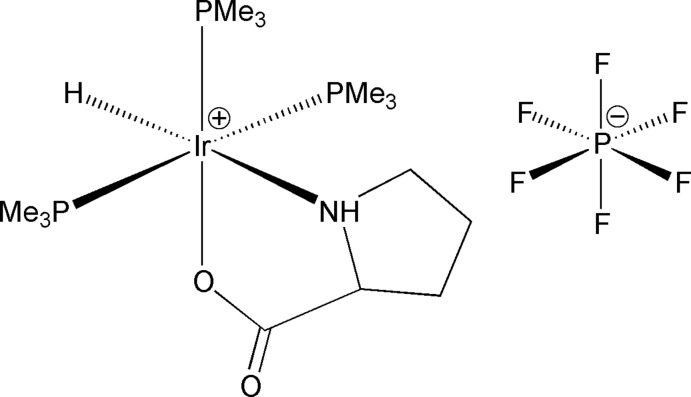


## Experimental   

### 

#### Crystal data  

[Ir(C_5_H_8_NO_2_)H(C_3_H_9_P)_3_]PF_6_
*M* *_r_* = 680.52
Monoclinic, 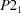

*a* = 10.824 (2) Å
*b* = 20.021 (4) Å
*c* = 11.826 (2) Å
β = 91.15 (1)°
*V* = 2562.3 (8) Å^3^
*Z* = 4
Mo *K*α radiation
μ = 5.51 mm^−1^
*T* = 293 K
0.5 × 0.4 × 0.4 mm

#### Data collection  

Siemens P4 diffractometer
Absorption correction: ψ scan (North *et al.*, 1968 ▶) *T*_min_ = 0.618, *T*_max_ = 1.000
6363 measured reflections
6055 independent reflections
5587 reflections with *I* > 2σ(*I*)
*R*_int_ = 0.020
3 standard reflections every 300 reflections intensity decay: none

#### Refinement  

*R*[*F* ^2^ > 2σ(*F* ^2^)] = 0.032
*wR*(*F* ^2^) = 0.075
*S* = 1.06
6055 reflections
522 parameters
4 restraints
H atoms treated by a mixture of independent and constrained refinement
Δρ_max_ = 1.02 e Å^−3^
Δρ_min_ = −0.93 e Å^−3^
Absolute structure: Flack (1983 ▶)
Absolute structure parameter: 0.001 (8)

### 

Data collection: *XSCANS* (Siemens, 1996 ▶); cell refinement: *XSCANS*; data reduction: *XSCANS*; program(s) used to solve structure: *SHELXS97* (Sheldrick, 2008 ▶); program(s) used to refine structure: *SHELXL97* (Sheldrick, 2008 ▶); molecular graphics: *OLEX2* (Dolomanov *et al.*, 2009 ▶); software used to prepare material for publication: *OLEX2*.

## Supplementary Material

Crystal structure: contains datablock(s) I. DOI: 10.1107/S1600536814001834/pk2511sup1.cif

Structure factors: contains datablock(s) I. DOI: 10.1107/S1600536814001834/pk2511Isup2.hkl

Supporting information file. DOI: 10.1107/S1600536814001834/pk2511Isup3.mol

CCDC reference:

Additional supporting information:  crystallographic information; 3D view; checkCIF report

## Figures and Tables

**Table 1:** Hydrogen-bond geometry (Å, °)

*D*—H⋯*A*	*D*—H	H⋯*A*	*D*⋯*A*	*D*—H⋯*A*
N1—H1⋯O4^i^	0.91	2.04	2.909 (9)	160

Symmetry code: (i) 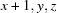.

**Fig. 1.**
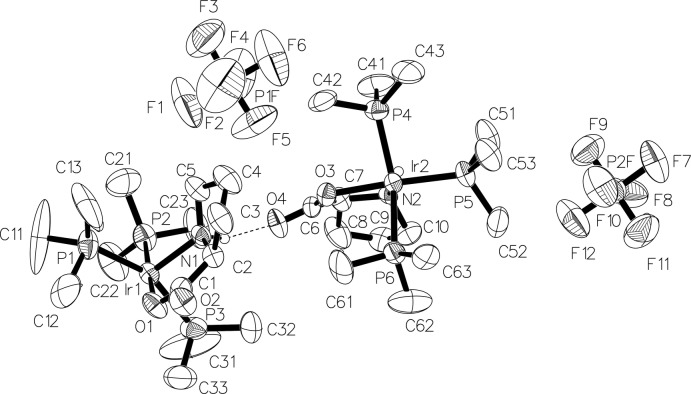
The asymmetric unit of the title compound, showing both sets of independent cations and anions. Hydrogen atoms are omitted for clarity. The displacement ellipsoids are shown at the 50% probability level and only the major component of the disordered methyl groups on P1 is shown.

**Fig. 2.**
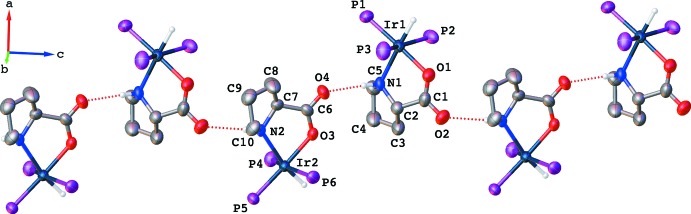
Hydrogen bonding motif for the title compound. Hydrogen atoms (with the exception of the metal hydrides and N—H atoms) and phosphorus methyl groups are omitted for clarity.) The displacement ellipsoids are shown at the 50% probability level.

## supplementary crystallographic information

### 2. Experimental

[Ir(COD)(PMe_3_)_3_]Cl was synthesized as reported previously (Frazier & Merola, 1992). L-proline was purchased from Aldrich Chemical and used as received. Water was deionized/distilled.

### 2.1. Synthesis and crystallization

A 100 mL flask equipped with a stir bar and septum was charged with 117.5 mg (1.02 mmol) of L-proline. The flask was then charged with 250 mg (0.443 mmol) of [Ir(COD)(PMe_3_)_3_]Cl under N_2_ in a drybox. The flask was then connected to a double manifold (vacuum/nitro­gen) Schlenk line and 20 mL of distilled water was added to the flask *via* syringe. The solution was stirred magnetically and heated to reflux. After 18 hours at reflux the reaction mixture was allowed to cool and solvent was removed *in vacuo*. The white solid residue was treated with distilled methyl­ene chloride (3 x 10 mL) to extract the product from the excess amino acid. The solution was filtered from the solid using cannula techniques. The methyl­ene chloride was removed *in vacuo* and the solids were dried under reduced pressure to yield 150 mg (0.263 mmol, 60.7% yield based on the amount of Ir(COD)(PMe_3_)_3_]Cl) of [Ir(L-pro)(H)(PMe_3_)_3_]Cl C,H analysis: Calculated for C_14_H_36_NO_2_P_3_IrCl:C, 29.4% H,6.3% Found: C, 29.17%; H, 6.2% An aqueous solution of the chloride salt was treated with an aqueous solution of K[PF_6_] to precipitate [Ir(L-pro)(H)(PMe_3_)_3_][PF_6_]. Crystals suitable for X-ray diffraction were grown from di­chloro­methane/di­ethyl­ether.

### 2.2. Refinement

Hydrogen atoms were treated with a combination of restrained refinement (*i.e.* for the Ir—H hydrogens) and constrained riding models. Values of U_iso_(H) were set to either 1.2U_eq_ or 1.5U_eq_ of the parent atom. Crystal data, data collection and structure refinement details are summarized in Table 1.

### 3. Results and discussion

The chemistry of amino acid complexes of transition metals has a long history (Shimazaki *et al.*, 2009). A search of the CSD data base (Allen, 2002) reveals over 1400 hits for compounds with bidentate N,O coordination of amino acids to transition metals. Restricting the search to the specific amino acid proline, yields over 140 hits. Finally, restricting the search to iridium compounds, there are 6 CSD structures with proline coordinated to iridium. We reported on the crystal structure of [hydrido-valinato-tris­(tri­methyl­phosphine)iridium][hexa­fluoro­phosphate] (CSD:20060907) (Roy *et al.*, 2006) in the space group P4_3_, which has very strong inter­molecular N—H···O hydrogen bonding that results in a 4_3_ helical arrangement of the iridium complexes. In this report, the title proline complex has a "flatter" inter­molecular motif with the N—H···O hydrogen bonding resulting in a one-dimenional chain motif with the chain parallel to the c-axis. Hydrogen bonding parameters are listed Table 1. A similar motif has been reported for (η^5^-Penta­methyl-cyclo­penta­dienyl)-(t-butyl­ethynyl)-(L-prolinato-N,O)-ιridium (CSD:20010919) (Carmona *et al.*, 2000), but when the t-butyl­ethynyl group is replaced by chloride (CSD: 19911024) (Carmona *et al.*, 2012), the motif changes to alternating N—H···O and N—H···Cl bonding to generate the full lattice.

### Crystal data

**Table:**

[Ir(C_5_H_8_NO_2_)H(C_3_H_9_P)_3_]PF_6_	*F*(000) = 1336
*M**_r_* = 680.52	*D*_x_ = 1.764 Mg m^−^^3^
Monoclinic, *P*2_1_	Mo *K*α radiation, λ = 0.71073 Å
*a* = 10.824 (2) Å	Cell parameters from 25 reflections
*b* = 20.021 (4) Å	θ = 2–22°
*c* = 11.826 (2) Å	µ = 5.51 mm^−^^1^
β = 91.15 (1)°	*T* = 293 K
*V* = 2562.3 (8) Å^3^	Prism, clear colourless
*Z* = 4	0.5 × 0.4 × 0.4 mm

### Data collection

**Table:**

Siemens P4 diffractometer	5587 reflections with *I* > 2σ(*I*)
Radiation source: fine-focus sealed tube	*R*_int_ = 0.020
Graphite monochromator	θ_max_ = 27.5°, θ_min_ = 1.9°
ω–scans	*h* = 0→14
Absorption correction: ψ scan (North *et al.*, 1968)	*k* = 0→26
*T*_min_ = 0.618, *T*_max_ = 1.000	*l* = −15→15
6363 measured reflections	3 standard reflections every 300 reflections
6055 independent reflections	intensity decay: 0(1)

### Refinement

**Table:**

Refinement on *F*^2^	Hydrogen site location: inferred from neighbouring sites
Least-squares matrix: full	H atoms treated by a mixture of independent and constrained refinement
*R*[*F*^2^ > 2σ(*F*^2^)] = 0.032	*w* = 1/[σ^2^(*F*_o_^2^) + (0.0445*P*)^2^ + 1.0877*P*] where *P* = (*F*_o_^2^ + 2*F*_c_^2^)/3
*wR*(*F*^2^) = 0.075	(Δ/σ)_max_ = 0.001
*S* = 1.06	Δρ_max_ = 1.02 e Å^−^^3^
6055 reflections	Δρ_min_ = −0.93 e Å^−^^3^
522 parameters	Extinction correction: *SHELXL97* (Sheldrick, 2008), Fc^*^=kFc[1+0.001xFc^2^λ^3^/sin(2θ)]^-1/4^
4 restraints	Extinction coefficient: 0.00105 (12)
Primary atom site location: structure-invariant direct methods	Absolute structure: Flack (1983)
Secondary atom site location: difference Fourier map	Absolute structure parameter: 0.001 (8)

### Special details

**Table:**

Geometry. All e.s.d.'s (except the e.s.d. in the dihedral angle between two l.s. planes) are estimated using the full covariance matrix. The cell e.s.d.'s are taken into account individually in the estimation of e.s.d.'s in distances, angles and torsion angles; correlations between e.s.d.'s in cell parameters are only used when they are defined by crystal symmetry. An approximate (isotropic) treatment of cell e.s.d.'s is used for estimating e.s.d.'s involving l.s. planes.
Refinement. Refinement of *F*^2^ against ALL reflections. The weighted *R*-factor *wR* and goodness of fit *S* are based on *F*^2^, conventional *R*-factors *R* are based on *F*, with *F* set to zero for negative *F*^2^. The threshold expression of *F*^2^ > σ(*F*^2^) is used only for calculating *R*-factors(gt) *etc*. and is not relevant to the choice of reflections for refinement. *R*-factors based on *F*^2^ are statistically about twice as large as those based on *F*, and *R*- factors based on ALL data will be even larger.

### Fractional atomic coordinates and isotropic or equivalent isotropic displacement parameters (Å^2^)

**Table:**

	*x*	*y*	*z*	*U*_iso_*/*U*_eq_	Occ. (<1)
Ir2	−0.08795 (2)	−0.490281 (13)	−0.05567 (2)	0.03453 (8)	
H	0.014 (7)	−0.486 (5)	0.021 (6)	0.041*	
P4	−0.0431 (2)	−0.38225 (14)	−0.1162 (2)	0.0514 (5)	
P5	0.0256 (2)	−0.54315 (14)	−0.18575 (19)	0.0491 (6)	
P6	−0.0981 (2)	−0.58276 (13)	0.0664 (2)	0.0500 (6)	
O3	−0.2047 (6)	−0.4449 (3)	0.0633 (5)	0.0438 (13)	
O4	−0.3980 (6)	−0.4334 (4)	0.1118 (5)	0.0622 (18)	
N2	−0.2684 (5)	−0.4939 (4)	−0.1474 (4)	0.0336 (12)	
H2A	−0.2590	−0.4713	−0.2134	0.040*	
C6	−0.3219 (8)	−0.4439 (4)	0.0432 (7)	0.0428 (18)	
C7	−0.3627 (8)	−0.4555 (5)	−0.0791 (7)	0.048 (2)	
H7	−0.3721	−0.4115	−0.1145	0.057*	
C8	−0.4842 (9)	−0.4914 (9)	−0.0947 (10)	0.082 (4)	
H8A	−0.5049	−0.5156	−0.0266	0.099*	
H8B	−0.5501	−0.4600	−0.1123	0.099*	
C9	−0.4661 (9)	−0.5386 (6)	−0.1910 (9)	0.065 (3)	
H9A	−0.4811	−0.5167	−0.2632	0.078*	
H9B	−0.5198	−0.5772	−0.1854	0.078*	
C10	−0.3311 (10)	−0.5583 (5)	−0.1762 (9)	0.055 (2)	
H10A	−0.3202	−0.5905	−0.1156	0.066*	
H10B	−0.2993	−0.5770	−0.2454	0.066*	
C41	−0.1356 (14)	−0.3456 (7)	−0.2278 (11)	0.092 (5)	
H41A	−0.1286	−0.3719	−0.2952	0.137*	
H41B	−0.1071	−0.3010	−0.2421	0.137*	
H41C	−0.2204	−0.3442	−0.2057	0.137*	
C42	−0.0602 (14)	−0.3248 (6)	0.0002 (10)	0.081 (4)	
H42A	−0.1433	−0.3268	0.0268	0.121*	
H42B	−0.0425	−0.2802	−0.0247	0.121*	
H42C	−0.0037	−0.3368	0.0605	0.121*	
C43	0.1141 (11)	−0.3655 (7)	−0.1608 (14)	0.096 (5)	
H43A	0.1710	−0.3748	−0.0995	0.144*	
H43B	0.1212	−0.3194	−0.1823	0.144*	
H43C	0.1330	−0.3934	−0.2241	0.144*	
C51	0.0109 (11)	−0.5114 (8)	−0.3276 (8)	0.081 (4)	
H51A	−0.0735	−0.5153	−0.3534	0.121*	
H51B	0.0631	−0.5366	−0.3764	0.121*	
H51C	0.0351	−0.4653	−0.3286	0.121*	
C52	−0.0078 (14)	−0.6317 (6)	−0.2063 (10)	0.081 (4)	
H52A	0.0053	−0.6550	−0.1361	0.122*	
H52B	0.0460	−0.6496	−0.2623	0.122*	
H52C	−0.0922	−0.6370	−0.2311	0.122*	
C53	0.1922 (9)	−0.5445 (8)	−0.1651 (11)	0.081 (4)	
H53A	0.2231	−0.4996	−0.1634	0.122*	
H53B	0.2291	−0.5684	−0.2262	0.122*	
H53C	0.2126	−0.5663	−0.0949	0.122*	
C61	−0.1468 (18)	−0.5558 (9)	0.2034 (10)	0.130 (8)	
H61A	−0.0897	−0.5232	0.2329	0.196*	
H61B	−0.1491	−0.5935	0.2535	0.196*	
H61C	−0.2277	−0.5364	0.1968	0.196*	
C62	−0.1973 (14)	−0.6552 (8)	0.0456 (15)	0.112 (6)	
H62A	−0.2823	−0.6415	0.0466	0.168*	
H62B	−0.1818	−0.6869	0.1051	0.168*	
H62C	−0.1803	−0.6755	−0.0260	0.168*	
C63	0.0501 (11)	−0.6209 (6)	0.0953 (9)	0.066 (3)	
H63A	0.0845	−0.6363	0.0258	0.099*	
H63B	0.0400	−0.6579	0.1457	0.099*	
H63C	0.1047	−0.5886	0.1297	0.099*	
Ir1	0.42609 (3)	−0.385828 (17)	0.42830 (3)	0.04158 (10)	
HA	0.291 (8)	−0.387 (5)	0.497 (7)	0.050*	
P1	0.4533 (3)	−0.29798 (17)	0.5578 (3)	0.0643 (8)	
P2	0.3190 (2)	−0.32928 (16)	0.2955 (2)	0.0585 (7)	
P3	0.3672 (3)	−0.49222 (17)	0.3689 (2)	0.0656 (7)	
O1	0.5350 (6)	−0.4376 (4)	0.5512 (5)	0.0592 (18)	
O2	0.7238 (7)	−0.4649 (4)	0.6036 (6)	0.068 (2)	
N1	0.6065 (6)	−0.3865 (4)	0.3446 (5)	0.0421 (14)	
H1	0.5944	−0.4097	0.2791	0.051*	
C1	0.6479 (10)	−0.4445 (5)	0.5318 (8)	0.057 (2)	
C2	0.6963 (8)	−0.4281 (5)	0.4158 (8)	0.048 (2)	
H2	0.7082	−0.4705	0.3761	0.057*	
C3	0.8210 (10)	−0.3902 (8)	0.4148 (8)	0.072 (3)	
H3A	0.8336	−0.3646	0.4837	0.087*	
H3B	0.8896	−0.4209	0.4062	0.087*	
C4	0.8077 (9)	−0.3443 (7)	0.3123 (9)	0.076 (3)	
H4A	0.8602	−0.3053	0.3203	0.092*	
H4B	0.8279	−0.3676	0.2431	0.092*	
C5	0.6716 (9)	−0.3252 (6)	0.3132 (9)	0.061 (3)	
H5A	0.6574	−0.2901	0.3680	0.073*	
H5B	0.6440	−0.3098	0.2392	0.073*	
C11	0.331 (3)	−0.243 (2)	0.585 (3)	0.23 (3)	0.62 (2)
H11A	0.2543	−0.2672	0.5831	0.339*	0.62 (2)
H11B	0.3284	−0.2084	0.5291	0.339*	0.62 (2)
H11C	0.3430	−0.2235	0.6588	0.339*	0.62 (2)
C12	0.448 (4)	−0.3406 (14)	0.7038 (19)	0.169 (17)	0.62 (2)
H12A	0.3827	−0.3731	0.7037	0.253*	0.62 (2)
H12B	0.4331	−0.3077	0.7609	0.253*	0.62 (2)
H12C	0.5254	−0.3624	0.7196	0.253*	0.62 (2)
C13	0.582 (3)	−0.252 (3)	0.579 (3)	0.23 (3)	0.62 (2)
H13A	0.6524	−0.2772	0.5561	0.351*	0.62 (2)
H13B	0.5899	−0.2407	0.6574	0.351*	0.62 (2)
H13C	0.5757	−0.2118	0.5346	0.351*	0.62 (2)
C11'	0.595 (4)	−0.281 (4)	0.620 (5)	0.23 (3)	0.38 (2)
H11D	0.6517	−0.2680	0.5624	0.339*	0.38 (2)
H11E	0.6253	−0.3202	0.6577	0.339*	0.38 (2)
H11F	0.5865	−0.2454	0.6732	0.339*	0.38 (2)
C12'	0.439 (7)	−0.204 (2)	0.497 (3)	0.169 (17)	0.38 (2)
H12D	0.4875	−0.2004	0.4302	0.253*	0.38 (2)
H12E	0.4690	−0.1735	0.5532	0.253*	0.38 (2)
H12F	0.3542	−0.1948	0.4788	0.253*	0.38 (2)
C13'	0.349 (5)	−0.296 (4)	0.648 (5)	0.23 (3)	0.38 (2)
H13D	0.2712	−0.3078	0.6125	0.351*	0.38 (2)
H13E	0.3435	−0.2520	0.6792	0.351*	0.38 (2)
H13F	0.3679	−0.3275	0.7072	0.351*	0.38 (2)
C21	0.3609 (18)	−0.2415 (7)	0.2790 (16)	0.115 (6)	
H21A	0.3479	−0.2183	0.3488	0.173*	
H21B	0.3105	−0.2218	0.2202	0.173*	
H21C	0.4463	−0.2382	0.2594	0.173*	
C22	0.1528 (11)	−0.3273 (10)	0.3072 (12)	0.102 (6)	
H22A	0.1212	−0.3721	0.3058	0.154*	
H22B	0.1175	−0.3025	0.2450	0.154*	
H22C	0.1314	−0.3062	0.3770	0.154*	
C23	0.3395 (14)	−0.3606 (10)	0.1514 (10)	0.104 (6)	
H23A	0.4253	−0.3583	0.1327	0.156*	
H23B	0.2920	−0.3338	0.0992	0.156*	
H23C	0.3119	−0.4061	0.1468	0.156*	
C31	0.220 (2)	−0.5030 (10)	0.303 (2)	0.194 (13)	
H31A	0.1568	−0.4896	0.3546	0.291*	
H31B	0.2083	−0.5491	0.2835	0.291*	
H31C	0.2142	−0.4761	0.2362	0.291*	
C32	0.465 (2)	−0.5422 (10)	0.2820 (17)	0.177 (12)	
H32A	0.4793	−0.5192	0.2124	0.265*	
H32B	0.4246	−0.5841	0.2661	0.265*	
H32C	0.5417	−0.5502	0.3210	0.265*	
C33	0.3521 (18)	−0.5449 (8)	0.4876 (13)	0.116 (6)	
H33A	0.4302	−0.5481	0.5272	0.173*	
H33B	0.3265	−0.5885	0.4630	0.173*	
H33C	0.2916	−0.5267	0.5372	0.173*	
P2F	0.9668 (3)	−0.18818 (16)	0.5529 (2)	0.0642 (7)	
F7	0.8585 (9)	−0.1880 (7)	0.6377 (7)	0.125 (4)	
F8	1.0611 (9)	−0.1994 (5)	0.6545 (7)	0.107 (3)	
F9	0.9816 (11)	−0.1118 (4)	0.5672 (10)	0.119 (3)	
F10	0.8701 (9)	−0.1759 (7)	0.4529 (6)	0.126 (4)	
F11	0.9558 (16)	−0.2645 (5)	0.5403 (9)	0.171 (6)	
F12	1.0760 (9)	−0.1859 (7)	0.4653 (8)	0.134 (4)	
P1F	0.5916 (3)	−0.20678 (17)	−0.0328 (3)	0.0726 (8)	
F1	0.4971 (14)	−0.1944 (10)	0.0534 (13)	0.206 (8)	
F2	0.6848 (16)	−0.2087 (9)	0.0641 (13)	0.218 (8)	
F3	0.6158 (16)	−0.1305 (6)	−0.0336 (11)	0.178 (6)	
F4	0.4963 (12)	−0.1990 (8)	−0.1305 (11)	0.184 (6)	
F5	0.5793 (17)	−0.2797 (6)	−0.0332 (18)	0.229 (8)	
F6	0.6855 (13)	−0.2143 (11)	−0.1255 (12)	0.214 (8)	

### Atomic displacement parameters (Å^2^)

**Table:**

	*U*^11^	*U*^22^	*U*^33^	*U*^12^	*U*^13^	*U*^23^
Ir2	0.03230 (13)	0.04134 (16)	0.02985 (13)	0.00099 (14)	−0.00191 (10)	−0.00124 (14)
P4	0.0558 (12)	0.0494 (13)	0.0487 (11)	−0.0107 (12)	−0.0023 (10)	0.0037 (12)
P5	0.0391 (11)	0.0700 (16)	0.0382 (11)	0.0085 (11)	0.0049 (9)	−0.0037 (11)
P6	0.0521 (12)	0.0526 (14)	0.0453 (12)	0.0123 (11)	0.0035 (10)	0.0121 (10)
O3	0.051 (3)	0.050 (3)	0.031 (3)	0.009 (3)	−0.005 (2)	−0.002 (2)
O4	0.059 (4)	0.090 (5)	0.038 (3)	0.011 (4)	0.011 (3)	−0.008 (3)
N2	0.041 (3)	0.039 (3)	0.022 (2)	−0.002 (3)	0.000 (2)	0.006 (3)
C6	0.045 (4)	0.041 (4)	0.043 (4)	0.007 (3)	0.009 (4)	−0.002 (4)
C7	0.038 (4)	0.062 (6)	0.044 (5)	0.010 (4)	0.000 (3)	0.004 (4)
C8	0.043 (5)	0.133 (11)	0.072 (7)	−0.017 (7)	0.010 (5)	−0.019 (9)
C9	0.056 (6)	0.076 (7)	0.064 (6)	−0.018 (5)	−0.013 (5)	0.000 (6)
C10	0.058 (6)	0.051 (5)	0.056 (5)	−0.011 (4)	−0.017 (5)	−0.007 (4)
C41	0.127 (12)	0.070 (8)	0.076 (8)	−0.018 (8)	−0.038 (8)	0.036 (7)
C42	0.135 (11)	0.043 (6)	0.065 (7)	−0.025 (7)	−0.001 (7)	−0.003 (5)
C43	0.065 (7)	0.079 (9)	0.145 (13)	−0.028 (6)	0.021 (8)	0.004 (8)
C51	0.070 (7)	0.132 (12)	0.040 (5)	0.021 (7)	0.005 (5)	−0.003 (6)
C52	0.108 (10)	0.069 (7)	0.066 (7)	0.024 (7)	0.009 (7)	−0.014 (6)
C53	0.036 (5)	0.124 (11)	0.084 (8)	0.017 (6)	0.007 (5)	0.005 (8)
C61	0.22 (2)	0.124 (13)	0.049 (7)	0.087 (14)	0.053 (10)	0.039 (8)
C62	0.084 (9)	0.095 (11)	0.156 (15)	−0.020 (8)	−0.030 (10)	0.064 (11)
C63	0.079 (7)	0.062 (6)	0.057 (6)	0.014 (6)	−0.014 (5)	0.014 (5)
Ir1	0.03547 (15)	0.0540 (2)	0.03524 (15)	−0.00449 (15)	0.00001 (12)	−0.00367 (15)
P1	0.0485 (13)	0.080 (2)	0.0641 (16)	0.0015 (13)	0.0004 (12)	−0.0302 (15)
P2	0.0446 (12)	0.0774 (18)	0.0532 (14)	0.0142 (13)	−0.0031 (11)	0.0018 (13)
P3	0.0754 (16)	0.0640 (16)	0.0574 (14)	−0.0267 (16)	−0.0038 (12)	−0.0042 (15)
O1	0.047 (4)	0.090 (5)	0.040 (3)	0.004 (4)	0.003 (3)	0.013 (3)
O2	0.061 (4)	0.090 (5)	0.053 (4)	0.015 (4)	−0.008 (3)	0.014 (4)
N1	0.048 (3)	0.052 (4)	0.026 (3)	−0.006 (4)	0.002 (2)	−0.001 (3)
C1	0.074 (7)	0.060 (6)	0.036 (4)	0.010 (5)	−0.008 (4)	−0.002 (4)
C2	0.041 (4)	0.058 (5)	0.043 (5)	−0.001 (4)	−0.001 (4)	−0.004 (4)
C3	0.054 (5)	0.119 (10)	0.044 (5)	0.002 (7)	−0.003 (4)	−0.006 (7)
C4	0.050 (5)	0.121 (10)	0.058 (6)	−0.024 (6)	0.003 (5)	−0.004 (7)
C5	0.050 (5)	0.080 (8)	0.054 (6)	−0.004 (5)	0.010 (4)	0.006 (5)
C11	0.18 (3)	0.33 (5)	0.17 (3)	0.20 (4)	−0.11 (3)	−0.18 (4)
C12	0.36 (5)	0.094 (17)	0.052 (11)	−0.04 (3)	0.02 (2)	−0.037 (11)
C13	0.14 (2)	0.38 (6)	0.18 (3)	−0.20 (4)	0.12 (3)	−0.21 (4)
C11'	0.18 (3)	0.33 (5)	0.17 (3)	0.20 (4)	−0.11 (3)	−0.18 (4)
C12'	0.36 (5)	0.094 (17)	0.052 (11)	−0.04 (3)	0.02 (2)	−0.037 (11)
C13'	0.14 (2)	0.38 (6)	0.18 (3)	−0.20 (4)	0.12 (3)	−0.21 (4)
C21	0.150 (16)	0.074 (9)	0.121 (13)	0.011 (10)	0.003 (12)	0.026 (9)
C22	0.048 (6)	0.174 (17)	0.085 (9)	0.020 (9)	−0.004 (6)	0.000 (10)
C23	0.091 (9)	0.171 (17)	0.050 (6)	0.040 (10)	−0.002 (6)	−0.011 (8)
C31	0.21 (2)	0.122 (16)	0.24 (3)	−0.112 (16)	−0.14 (2)	0.047 (16)
C32	0.28 (3)	0.100 (13)	0.154 (17)	−0.047 (16)	0.146 (19)	−0.062 (13)
C33	0.175 (17)	0.086 (10)	0.087 (10)	−0.052 (12)	0.031 (11)	0.000 (8)
P2F	0.088 (2)	0.0630 (17)	0.0421 (13)	−0.0072 (15)	0.0048 (13)	−0.0013 (12)
F7	0.109 (6)	0.198 (11)	0.068 (5)	−0.021 (7)	0.022 (4)	0.017 (6)
F8	0.127 (7)	0.121 (7)	0.072 (5)	0.039 (6)	−0.018 (5)	−0.018 (5)
F9	0.146 (9)	0.072 (6)	0.140 (9)	−0.009 (5)	0.003 (7)	−0.001 (5)
F10	0.122 (7)	0.193 (11)	0.061 (4)	−0.004 (8)	−0.023 (5)	0.004 (6)
F11	0.333 (19)	0.079 (6)	0.101 (7)	−0.045 (9)	0.018 (9)	−0.022 (5)
F12	0.118 (7)	0.211 (12)	0.075 (5)	0.047 (8)	0.031 (5)	0.026 (7)
P1F	0.078 (2)	0.072 (2)	0.0678 (18)	0.0101 (16)	0.0070 (16)	−0.0079 (15)
F1	0.171 (12)	0.28 (2)	0.167 (12)	0.026 (14)	0.099 (11)	−0.041 (13)
F2	0.253 (18)	0.223 (18)	0.174 (13)	0.044 (14)	−0.115 (13)	−0.024 (12)
F3	0.300 (18)	0.090 (7)	0.144 (10)	−0.004 (9)	−0.012 (11)	−0.020 (7)
F4	0.150 (10)	0.220 (15)	0.180 (12)	0.042 (10)	−0.080 (9)	−0.062 (11)
F5	0.30 (2)	0.072 (7)	0.32 (2)	−0.035 (11)	0.055 (17)	−0.006 (11)
F6	0.152 (11)	0.34 (2)	0.148 (11)	0.029 (13)	0.051 (9)	−0.060 (13)

### Geometric parameters (Å, º)

**Table:**

Ir2—H	1.42 (8)	P2—C21	1.827 (15)
Ir2—P4	2.332 (3)	P2—C22	1.807 (12)
Ir2—P5	2.252 (2)	P2—C23	1.833 (12)
Ir2—P6	2.352 (2)	P3—C31	1.774 (17)
Ir2—O3	2.114 (6)	P3—C32	1.792 (16)
Ir2—N2	2.216 (6)	P3—C33	1.766 (15)
P4—C41	1.798 (12)	O1—C1	1.256 (13)
P4—C42	1.807 (12)	O2—C1	1.238 (11)
P4—C43	1.823 (11)	N1—H1	0.9100
P5—C51	1.798 (11)	N1—C2	1.520 (11)
P5—C52	1.824 (13)	N1—C5	1.467 (13)
P5—C53	1.816 (10)	C1—C2	1.515 (13)
P6—C61	1.796 (12)	C2—H2	0.9800
P6—C62	1.819 (15)	C2—C3	1.548 (14)
P6—C63	1.802 (11)	C3—H3A	0.9700
O3—C6	1.287 (10)	C3—H3B	0.9700
O4—C6	1.186 (10)	C3—C4	1.525 (17)
N2—H2A	0.9100	C4—H4A	0.9700
N2—C7	1.521 (10)	C4—H4B	0.9700
N2—C10	1.493 (12)	C4—C5	1.522 (14)
C6—C7	1.522 (12)	C5—H5A	0.9700
C7—H7	0.9800	C5—H5B	0.9700
C7—C8	1.507 (14)	C11—H11A	0.9600
C8—H8A	0.9700	C11—H11B	0.9600
C8—H8B	0.9700	C11—H11C	0.9600
C8—C9	1.496 (17)	C12—H12A	0.9600
C9—H9A	0.9700	C12—H12B	0.9600
C9—H9B	0.9700	C12—H12C	0.9600
C9—C10	1.520 (15)	C13—H13A	0.9600
C10—H10A	0.9700	C13—H13B	0.9600
C10—H10B	0.9700	C13—H13C	0.9600
C41—H41A	0.9600	C11'—H11D	0.9600
C41—H41B	0.9600	C11'—H11E	0.9600
C41—H41C	0.9600	C11'—H11F	0.9600
C42—H42A	0.9600	C12'—H12D	0.9600
C42—H42B	0.9600	C12'—H12E	0.9600
C42—H42C	0.9600	C12'—H12F	0.9600
C43—H43A	0.9600	C13'—H13D	0.9600
C43—H43B	0.9600	C13'—H13E	0.9600
C43—H43C	0.9600	C13'—H13F	0.9600
C51—H51A	0.9600	C21—H21A	0.9600
C51—H51B	0.9600	C21—H21B	0.9600
C51—H51C	0.9600	C21—H21C	0.9600
C52—H52A	0.9600	C22—H22A	0.9600
C52—H52B	0.9600	C22—H22B	0.9600
C52—H52C	0.9600	C22—H22C	0.9600
C53—H53A	0.9600	C23—H23A	0.9600
C53—H53B	0.9600	C23—H23B	0.9600
C53—H53C	0.9600	C23—H23C	0.9600
C61—H61A	0.9600	C31—H31A	0.9600
C61—H61B	0.9600	C31—H31B	0.9600
C61—H61C	0.9600	C31—H31C	0.9600
C62—H62A	0.9600	C32—H32A	0.9600
C62—H62B	0.9600	C32—H32B	0.9600
C62—H62C	0.9600	C32—H32C	0.9600
C63—H63A	0.9600	C33—H33A	0.9600
C63—H63B	0.9600	C33—H33B	0.9600
C63—H63C	0.9600	C33—H33C	0.9600
Ir1—HA	1.69 (8)	P2F—F7	1.559 (8)
Ir1—P1	2.346 (3)	P2F—F8	1.577 (8)
Ir1—P2	2.241 (3)	P2F—F9	1.545 (9)
Ir1—P3	2.328 (3)	P2F—F10	1.583 (8)
Ir1—O1	2.123 (7)	P2F—F11	1.539 (10)
Ir1—N1	2.206 (6)	P2F—F12	1.588 (9)
P1—C11	1.76 (2)	P1F—F1	1.480 (11)
P1—C12	1.93 (3)	P1F—F2	1.512 (13)
P1—C13	1.68 (2)	P1F—F3	1.550 (13)
P1—C11'	1.72 (5)	P1F—F4	1.540 (11)
P1—C12'	2.01 (4)	P1F—F5	1.466 (12)
P1—C13'	1.57 (4)	P1F—F6	1.517 (12)
			
P4—Ir2—H	89 (4)	C13'—P1—C12	51 (3)
P4—Ir2—P6	158.58 (9)	C13'—P1—C13	119.4 (17)
P5—Ir2—H	92 (3)	C13'—P1—C11'	111 (2)
P5—Ir2—P4	96.12 (10)	C13'—P1—C12'	100 (3)
P5—Ir2—P6	94.71 (9)	C21—P2—Ir1	115.8 (6)
P6—Ir2—H	72 (4)	C21—P2—C23	101.3 (9)
O3—Ir2—H	91 (3)	C22—P2—Ir1	117.3 (5)
O3—Ir2—P4	86.36 (18)	C22—P2—C21	103.7 (9)
O3—Ir2—P5	176.11 (18)	C22—P2—C23	102.6 (7)
O3—Ir2—P6	83.91 (17)	C23—P2—Ir1	114.1 (5)
O3—Ir2—N2	78.9 (2)	C31—P3—Ir1	118.8 (7)
N2—Ir2—H	169 (3)	C31—P3—C32	102.5 (12)
N2—Ir2—P4	93.83 (19)	C32—P3—Ir1	121.6 (7)
N2—Ir2—P5	97.94 (17)	C33—P3—Ir1	109.6 (5)
N2—Ir2—P6	102.91 (18)	C33—P3—C31	100.3 (9)
C41—P4—Ir2	119.2 (4)	C33—P3—C32	100.8 (10)
C41—P4—C42	103.6 (7)	C1—O1—Ir1	117.2 (6)
C41—P4—C43	102.8 (7)	Ir1—N1—H1	105.8
C42—P4—Ir2	109.4 (4)	C2—N1—Ir1	108.5 (5)
C42—P4—C43	102.4 (7)	C2—N1—H1	105.8
C43—P4—Ir2	117.4 (5)	C5—N1—Ir1	122.8 (6)
C51—P5—Ir2	115.6 (4)	C5—N1—H1	105.8
C51—P5—C52	101.9 (6)	C5—N1—C2	107.0 (7)
C51—P5—C53	101.5 (6)	O1—C1—C2	119.8 (8)
C52—P5—Ir2	116.0 (4)	O2—C1—O1	123.1 (9)
C53—P5—Ir2	118.0 (5)	O2—C1—C2	117.1 (10)
C53—P5—C52	101.3 (7)	N1—C2—H2	107.3
C61—P6—Ir2	109.6 (5)	N1—C2—C3	106.0 (8)
C61—P6—C62	100.3 (9)	C1—C2—N1	113.0 (7)
C61—P6—C63	103.4 (7)	C1—C2—H2	107.3
C62—P6—Ir2	125.4 (5)	C1—C2—C3	115.5 (8)
C63—P6—Ir2	113.4 (4)	C3—C2—H2	107.3
C63—P6—C62	102.1 (6)	C2—C3—H3A	111.1
C6—O3—Ir2	119.0 (5)	C2—C3—H3B	111.1
Ir2—N2—H2A	106.9	H3A—C3—H3B	109.0
C7—N2—Ir2	108.5 (4)	C4—C3—C2	103.4 (8)
C7—N2—H2A	106.9	C4—C3—H3A	111.1
C10—N2—Ir2	122.1 (6)	C4—C3—H3B	111.1
C10—N2—H2A	106.9	C3—C4—H4A	111.2
C10—N2—C7	104.5 (7)	C3—C4—H4B	111.2
O3—C6—C7	116.0 (7)	H4A—C4—H4B	109.1
O4—C6—O3	125.0 (9)	C5—C4—C3	102.8 (8)
O4—C6—C7	119.0 (8)	C5—C4—H4A	111.2
N2—C7—C6	113.4 (6)	C5—C4—H4B	111.2
N2—C7—H7	107.1	N1—C5—C4	105.1 (9)
C6—C7—H7	107.1	N1—C5—H5A	110.7
C8—C7—N2	106.7 (8)	N1—C5—H5B	110.7
C8—C7—C6	115.1 (8)	C4—C5—H5A	110.7
C8—C7—H7	107.1	C4—C5—H5B	110.7
C7—C8—H8A	110.7	H5A—C5—H5B	108.8
C7—C8—H8B	110.7	P1—C11—H11A	109.5
H8A—C8—H8B	108.8	P1—C11—H11B	109.5
C9—C8—C7	105.4 (8)	P1—C11—H11C	109.5
C9—C8—H8A	110.7	H11A—C11—H11B	109.5
C9—C8—H8B	110.7	H11A—C11—H11C	109.5
C8—C9—H9A	111.3	H11B—C11—H11C	109.5
C8—C9—H9B	111.3	P1—C12—H12A	109.5
C8—C9—C10	102.5 (8)	P1—C12—H12B	109.5
H9A—C9—H9B	109.2	P1—C12—H12C	109.5
C10—C9—H9A	111.3	H12A—C12—H12B	109.5
C10—C9—H9B	111.3	H12A—C12—H12C	109.5
N2—C10—C9	103.5 (8)	H12B—C12—H12C	109.5
N2—C10—H10A	111.1	P1—C13—H13A	109.5
N2—C10—H10B	111.1	P1—C13—H13B	109.5
C9—C10—H10A	111.1	P1—C13—H13C	109.5
C9—C10—H10B	111.1	H13A—C13—H13B	109.5
H10A—C10—H10B	109.0	H13A—C13—H13C	109.5
P4—C41—H41A	109.5	H13B—C13—H13C	109.5
P4—C41—H41B	109.5	P1—C11'—H11D	109.5
P4—C41—H41C	109.5	P1—C11'—H11E	109.5
H41A—C41—H41B	109.5	P1—C11'—H11F	109.5
H41A—C41—H41C	109.5	H11D—C11'—H11E	109.5
H41B—C41—H41C	109.5	H11D—C11'—H11F	109.5
P4—C42—H42A	109.5	H11E—C11'—H11F	109.5
P4—C42—H42B	109.5	P1—C12'—H12D	109.5
P4—C42—H42C	109.5	P1—C12'—H12E	109.5
H42A—C42—H42B	109.5	P1—C12'—H12F	109.5
H42A—C42—H42C	109.5	H12D—C12'—H12E	109.5
H42B—C42—H42C	109.5	H12D—C12'—H12F	109.5
P4—C43—H43A	109.5	H12E—C12'—H12F	109.5
P4—C43—H43B	109.5	P1—C13'—H13D	109.5
P4—C43—H43C	109.5	P1—C13'—H13E	109.5
H43A—C43—H43B	109.5	P1—C13'—H13F	109.5
H43A—C43—H43C	109.5	H13D—C13'—H13E	109.5
H43B—C43—H43C	109.5	H13D—C13'—H13F	109.5
P5—C51—H51A	109.5	H13E—C13'—H13F	109.5
P5—C51—H51B	109.5	P2—C21—H21A	109.5
P5—C51—H51C	109.5	P2—C21—H21B	109.5
H51A—C51—H51B	109.5	P2—C21—H21C	109.5
H51A—C51—H51C	109.5	H21A—C21—H21B	109.5
H51B—C51—H51C	109.5	H21A—C21—H21C	109.5
P5—C52—H52A	109.5	H21B—C21—H21C	109.5
P5—C52—H52B	109.5	P2—C22—H22A	109.5
P5—C52—H52C	109.5	P2—C22—H22B	109.5
H52A—C52—H52B	109.5	P2—C22—H22C	109.5
H52A—C52—H52C	109.5	H22A—C22—H22B	109.5
H52B—C52—H52C	109.5	H22A—C22—H22C	109.5
P5—C53—H53A	109.5	H22B—C22—H22C	109.5
P5—C53—H53B	109.5	P2—C23—H23A	109.5
P5—C53—H53C	109.5	P2—C23—H23B	109.5
H53A—C53—H53B	109.5	P2—C23—H23C	109.5
H53A—C53—H53C	109.5	H23A—C23—H23B	109.5
H53B—C53—H53C	109.5	H23A—C23—H23C	109.5
P6—C61—H61A	109.5	H23B—C23—H23C	109.5
P6—C61—H61B	109.5	P3—C31—H31A	109.5
P6—C61—H61C	109.5	P3—C31—H31B	109.5
H61A—C61—H61B	109.5	P3—C31—H31C	109.5
H61A—C61—H61C	109.5	H31A—C31—H31B	109.5
H61B—C61—H61C	109.5	H31A—C31—H31C	109.5
P6—C62—H62A	109.5	H31B—C31—H31C	109.5
P6—C62—H62B	109.5	P3—C32—H32A	109.5
P6—C62—H62C	109.5	P3—C32—H32B	109.5
H62A—C62—H62B	109.5	P3—C32—H32C	109.5
H62A—C62—H62C	109.5	H32A—C32—H32B	109.5
H62B—C62—H62C	109.5	H32A—C32—H32C	109.5
P6—C63—H63A	109.5	H32B—C32—H32C	109.5
P6—C63—H63B	109.5	P3—C33—H33A	109.5
P6—C63—H63C	109.5	P3—C33—H33B	109.5
H63A—C63—H63B	109.5	P3—C33—H33C	109.5
H63A—C63—H63C	109.5	H33A—C33—H33B	109.5
H63B—C63—H63C	109.5	H33A—C33—H33C	109.5
P1—Ir1—HA	78 (3)	H33B—C33—H33C	109.5
P2—Ir1—HA	84 (3)	F7—P2F—F8	89.6 (5)
P2—Ir1—P1	97.77 (12)	F7—P2F—F10	89.2 (5)
P2—Ir1—P3	96.65 (11)	F7—P2F—F12	178.1 (7)
P3—Ir1—HA	84 (3)	F8—P2F—F10	178.6 (6)
P3—Ir1—P1	155.90 (11)	F8—P2F—F12	91.3 (5)
O1—Ir1—HA	98 (3)	F9—P2F—F7	90.3 (7)
O1—Ir1—P1	81.9 (2)	F9—P2F—F8	89.6 (6)
O1—Ir1—P2	177.4 (2)	F9—P2F—F10	89.7 (7)
O1—Ir1—P3	84.5 (2)	F9—P2F—F12	88.0 (7)
O1—Ir1—N1	79.6 (2)	F10—P2F—F12	89.9 (5)
N1—Ir1—HA	178 (3)	F11—P2F—F7	90.4 (7)
N1—Ir1—P1	101.4 (2)	F11—P2F—F8	88.8 (7)
N1—Ir1—P2	97.99 (18)	F11—P2F—F9	178.3 (8)
N1—Ir1—P3	95.6 (2)	F11—P2F—F10	91.9 (8)
C11—P1—Ir1	120.3 (11)	F11—P2F—F12	91.3 (8)
C11—P1—C12	94.3 (17)	F1—P1F—F2	86.6 (10)
C11—P1—C12'	55 (2)	F1—P1F—F3	87.6 (9)
C12—P1—Ir1	104.3 (8)	F1—P1F—F4	92.2 (9)
C12—P1—C12'	136.8 (13)	F1—P1F—F6	175.5 (11)
C13—P1—Ir1	126.8 (11)	F2—P1F—F3	85.3 (9)
C13—P1—C11	105 (2)	F2—P1F—F4	175.6 (9)
C13—P1—C12	98.6 (17)	F2—P1F—F6	95.7 (10)
C13—P1—C12'	66 (2)	F4—P1F—F3	90.4 (8)
C11'—P1—Ir1	121.7 (15)	F5—P1F—F1	96.0 (11)
C11'—P1—C11	117.8 (18)	F5—P1F—F2	92.1 (11)
C11'—P1—C12	75 (3)	F5—P1F—F3	175.5 (10)
C11'—P1—C12'	92 (2)	F5—P1F—F4	92.2 (10)
C12'—P1—Ir1	117.2 (11)	F5—P1F—F6	87.8 (11)
C13'—P1—Ir1	112.2 (17)	F6—P1F—F3	88.8 (10)
C13'—P1—C11	46 (3)	F6—P1F—F4	85.3 (8)
			
Ir2—O3—C6—O4	162.9 (8)	P1—Ir1—P2—C22	−88.9 (7)
Ir2—O3—C6—C7	−18.6 (10)	P1—Ir1—P2—C23	151.1 (7)
Ir2—N2—C7—C6	−17.1 (9)	P1—Ir1—P3—C31	95.9 (10)
Ir2—N2—C7—C8	−144.8 (7)	P1—Ir1—P3—C32	−135.3 (10)
Ir2—N2—C10—C9	157.3 (6)	P1—Ir1—P3—C33	−18.5 (8)
P4—Ir2—P5—C51	−44.0 (5)	P1—Ir1—O1—C1	−100.2 (8)
P4—Ir2—P5—C52	−163.2 (5)	P1—Ir1—N1—C2	84.8 (5)
P4—Ir2—P5—C53	76.3 (6)	P1—Ir1—N1—C5	−40.8 (7)
P4—Ir2—P6—C61	44.0 (8)	P2—Ir1—P1—C11	42 (2)
P4—Ir2—P6—C62	163.1 (8)	P2—Ir1—P1—C12	145.8 (15)
P4—Ir2—P6—C63	−71.0 (5)	P2—Ir1—P1—C13	−102 (3)
P4—Ir2—O3—C6	101.2 (6)	P2—Ir1—P1—C11'	−133 (3)
P4—Ir2—N2—C7	−79.0 (5)	P2—Ir1—P1—C12'	−22 (2)
P4—Ir2—N2—C10	159.5 (6)	P2—Ir1—P1—C13'	93 (4)
P5—Ir2—P4—C41	86.2 (6)	P2—Ir1—P3—C31	−30.6 (10)
P5—Ir2—P4—C42	−155.0 (5)	P2—Ir1—P3—C32	98.3 (10)
P5—Ir2—P4—C43	−39.0 (6)	P2—Ir1—P3—C33	−144.9 (7)
P5—Ir2—P6—C61	164.2 (7)	P2—Ir1—N1—C2	−175.6 (5)
P5—Ir2—P6—C62	−76.8 (8)	P2—Ir1—N1—C5	58.8 (7)
P5—Ir2—P6—C63	49.2 (4)	P3—Ir1—P1—C11	−84 (2)
P5—Ir2—N2—C7	−175.7 (5)	P3—Ir1—P1—C12	19.5 (15)
P5—Ir2—N2—C10	62.8 (6)	P3—Ir1—P1—C13	132 (3)
P6—Ir2—P4—C41	−153.9 (6)	P3—Ir1—P1—C11'	101 (3)
P6—Ir2—P4—C42	−35.0 (6)	P3—Ir1—P1—C12'	−148 (2)
P6—Ir2—P4—C43	80.9 (6)	P3—Ir1—P1—C13'	−34 (4)
P6—Ir2—P5—C51	154.5 (5)	P3—Ir1—P2—C21	−165.2 (7)
P6—Ir2—P5—C52	35.3 (5)	P3—Ir1—P2—C22	71.7 (7)
P6—Ir2—P5—C53	−85.2 (6)	P3—Ir1—P2—C23	−48.3 (7)
P6—Ir2—O3—C6	−97.9 (6)	P3—Ir1—O1—C1	99.7 (7)
P6—Ir2—N2—C7	87.6 (5)	P3—Ir1—N1—C2	−78.0 (5)
P6—Ir2—N2—C10	−33.9 (6)	P3—Ir1—N1—C5	156.3 (6)
O3—Ir2—P4—C41	−90.8 (7)	O1—Ir1—P1—C11	−141 (2)
O3—Ir2—P4—C42	28.0 (6)	O1—Ir1—P1—C12	−36.8 (15)
O3—Ir2—P4—C43	144.0 (6)	O1—Ir1—P1—C13	76 (3)
O3—Ir2—P6—C61	−19.4 (7)	O1—Ir1—P1—C11'	45 (3)
O3—Ir2—P6—C62	99.6 (8)	O1—Ir1—P1—C12'	156 (2)
O3—Ir2—P6—C63	−134.5 (5)	O1—Ir1—P1—C13'	−90 (4)
O3—Ir2—N2—C7	6.5 (5)	O1—Ir1—P3—C31	151.7 (10)
O3—Ir2—N2—C10	−115.0 (6)	O1—Ir1—P3—C32	−79.5 (10)
O3—C6—C7—N2	23.9 (11)	O1—Ir1—P3—C33	37.4 (7)
O3—C6—C7—C8	147.2 (10)	O1—Ir1—N1—C2	5.4 (5)
O4—C6—C7—N2	−157.5 (8)	O1—Ir1—N1—C5	−120.3 (7)
O4—C6—C7—C8	−34.2 (14)	O1—C1—C2—N1	16.0 (13)
N2—Ir2—P4—C41	−12.3 (7)	O1—C1—C2—C3	138.4 (11)
N2—Ir2—P4—C42	106.6 (6)	O2—C1—C2—N1	−164.5 (9)
N2—Ir2—P4—C43	−137.4 (6)	O2—C1—C2—C3	−42.1 (14)
N2—Ir2—P5—C51	50.8 (6)	N1—Ir1—P1—C11	142 (2)
N2—Ir2—P5—C52	−68.4 (5)	N1—Ir1—P1—C12	−114.4 (15)
N2—Ir2—P5—C53	171.1 (6)	N1—Ir1—P1—C13	−2 (3)
N2—Ir2—P6—C61	−96.5 (7)	N1—Ir1—P1—C11'	−33 (3)
N2—Ir2—P6—C62	22.5 (8)	N1—Ir1—P1—C12'	78 (2)
N2—Ir2—P6—C63	148.5 (5)	N1—Ir1—P1—C13'	−167 (4)
N2—Ir2—O3—C6	6.6 (6)	N1—Ir1—P2—C21	−68.6 (7)
N2—C7—C8—C9	−13.1 (13)	N1—Ir1—P2—C22	168.4 (7)
C6—C7—C8—C9	−139.9 (10)	N1—Ir1—P2—C23	48.4 (7)
C7—N2—C10—C9	33.9 (9)	N1—Ir1—P3—C31	−129.3 (10)
C7—C8—C9—C10	33.7 (13)	N1—Ir1—P3—C32	−0.5 (10)
C8—C9—C10—N2	−42.1 (11)	N1—Ir1—P3—C33	116.4 (7)
C10—N2—C7—C6	114.7 (8)	N1—Ir1—O1—C1	3.0 (7)
C10—N2—C7—C8	−13.1 (10)	N1—C2—C3—C4	−19.3 (11)
Ir1—O1—C1—O2	169.2 (8)	C1—C2—C3—C4	−145.3 (9)
Ir1—O1—C1—C2	−11.3 (12)	C2—N1—C5—C4	27.6 (10)
Ir1—N1—C2—C1	−11.9 (9)	C2—C3—C4—C5	35.5 (12)
Ir1—N1—C2—C3	−139.4 (6)	C3—C4—C5—N1	−39.7 (11)
Ir1—N1—C5—C4	153.9 (6)	C5—N1—C2—C1	122.5 (8)
P1—Ir1—P2—C21	34.1 (7)	C5—N1—C2—C3	−5.0 (9)

### Hydrogen-bond geometry (Å, º)

**Table:**

*D*—H···*A*	*D*—H	H···*A*	*D*···*A*	*D*—H···*A*
N1—H1···O4^i^	0.91	2.04	2.909 (9)	160

Symmetry code: (i) *x*+1, *y*, *z*.
